# Symptom improvement in children with autism spectrum disorder following bumetanide administration is associated with decreased GABA/glutamate ratios

**DOI:** 10.1038/s41398-020-0692-2

**Published:** 2020-01-27

**Authors:** Lingli Zhang, Chu-Chung Huang, Yuan Dai, Qiang Luo, Yiting Ji, Kai Wang, Shining Deng, Juehua Yu, Mingyu Xu, Xiujuan Du, Yun Tang, Chun Shen, Jianfeng Feng, Barbara J Sahakian, Ching-Po Lin, Fei Li

**Affiliations:** 1 Developmental and Behavioral Pediatric Department & Child Primary Care Department, Brain and Behavioral Research Unit of Shanghai Institute for Pediatric Research and MOE- Shanghai Key Laboratory for Children’s Environmental Health, Xinhua Hospital, Shanghai Jiao Tong University School of Medicine, Shanghai, China; 2 Shanghai Institute of Pediatric Research, Xinhua Hospital, Shanghai Jiao Tong University School of Medicine, Shanghai, China; 3 Institute of Science and Technology for Brain-Inspired Intelligence, MOE-Key Laboratory of Computational Neuroscience and Brain-Inspired Intelligence, Fudan University, Shanghai, China; 4 State Key Laboratory of Medical Neurobiology and MOE Frontiers Center for Brain Science, Institute of Brain Science and Human Phenom Institute, Fudan University, Shanghai, China; 5 Departments of Psychology and Psychiatry and the Behavioural and Clinical Neuroscience Institute, University of Cambridge, Cambridge, UK; 6 Institute of Neuroscience, National Yang-Ming University, Taipei, Taiwan

**Keywords:** Predictive markers, Autism spectrum disorders

## Abstract

Bumetanide has been reported to alter synaptic excitation–inhibition (E-I) balance by potentiating the action of γ-aminobutyric acid (GABA), thereby attenuating the severity of autism spectrum disorder (ASD) in animal models. However, clinical evidence of its efficacy in young patients with ASD is limited. This was investigated in the present clinical trial of 83 patients, randomised to the bumetanide group (bumetanide treatment, 0.5 mg twice daily) or the control group (no bumetanide treatment). Primary [Children Autism Rating Scale (CARS)], secondary [Clinical Global Impressions (CGI)], and exploratory [inhibitory (γ-aminobutyric acid, GABA) and excitatory (glutamate, Glx) neurotransmitter concentrations measured in the insular cortex (IC) and visual cortex (VC) by magnetic resonance spectroscopy (MRS)] outcome measures were evaluated at baseline and at the 3-month follow-up. Side effects were monitored throughout the treatment course. Compared with the control group, the bumetanide group showed significant reduction in symptom severity, as indicated by both total CARS score and number of items assigned a score ≥ 3. The improvement in clinical symptoms was confirmed by CGI. GABA/Glx ratio in both the IC and VC decreased more rapidly over the 3-month period in the bumetanide group than that in the control group. This decrease in the IC was associated with the symptom improvement in the bumetanide group. Our study confirmed the clinical efficacy of bumetanide on alleviating the core symptoms of ASD in young children and it is the first demonstration that the improvement is associated with reduction in GABA/Glx ratios. This study suggests that the GABA/Glx ratio measured by MRS may provide a neuroimaging biomarker for assessing treatment efficacy for bumetanide.

## Introduction

Autism spectrum disorder (ASD) is a neurodevelopmental disorder with an increasing global prevalence ranging from 42.6/10,000 in China to 1/58 in the United States^1,2^. ASD can be reliably diagnosed at 24 months or even as early as 18 months of age^3^, and is a life-long condition. Although the molecular and neural mechanisms underlying ASD remain largely unknown, a previous study suggested that ASD may result from alteration in brain development during early life, such as the excitatory-inhibitory (E-I) imbalance in the autistic brain can affect the sensory, memory and emotional systems^4^, indicating the necessity of early intervention. Without directly monitoring the neuronal E-I balance, current behavioural interventions for ASD at preschool age are mainly behavioural intervention, including parents-mediate Early Start Denver Model and the Applied Behaviour Analysis^5^. These intervention resources vary greatly between countries and within regions, for example, less well-educated families in Europe may not receive behavioural intervention for ASD children for a year following diagnosis^6^. Furthermore, this failure to receive behavioural treatment is even worse in developing countries^7^. Therefore, a pharmacological treatment remains an alternative approach, which could be used globally where other behavioural treatments are not readily available. However, risperidone and aripiprazole, the only medications for ASD approved by the U.S. Food and Drug Administration, do not attenuate ASD core symptoms, and have considerable adverse side effects^5^. Therefore, there is a need for treatment strategies focused on the atypical early development of the autistic brain that can mitigate ASD symptoms, including impairments in social and emotional cognition.

The E-I imbalance has been hypothesized to be caused by an unsuccessful excitatory-to-inhibitory shift of the GABA (γ-aminobutyric acid) activity in neurons, known as the GABA switch^8^, and this switch may depend on a reduction of intracellular chloride concentration ([Cl^–^]_i_) mediated by a sequential expression of the main chloride transporters, especially the importer Na-K-Cl cotransporter 1 (NKCC1)^9^. The regulation of the intracellular neuronal chloride levels determines the efficacy of GABAergic inhibition and high levels can hinder the polarity from excitation to inhibition^10^. In animal models of autism, notably rats with intrauterine valproic acid injection^11,12^ or maternal immune activation^13^, rats carrying the fragile X^11^ or MECP2 mutation^14^, acute maternal administration of bumetanide, a NKCC1 chloride-importer inhibitor, before delivery switched the action of GABA from excitatory to inhibitory in the offspring, restoring both electrophysiological profile of the CA3 area of hippocampus, cerebellar purkinje cells or normal cerebral volumes.

Recent clinical trials have shown that bumetanide can reduce the severity of autism with effect sizes ranging from 0.33 to 0.64 and only bring few adverse events^10,15–17^. Similar clinical improvement as well as EEG alterations, have been observed in an ASD girl with 15q11.2 duplication after bumetanide treatment^18^. A more recent report revealed that bumetanide could reduce amygdala activation in adolescents with ASD, in which authors hypothesized that bumetanide restores the E-I balance of brain^19^. However, no studies to date have directly tested whether bumetanide regulate E-I balance by facilitating the GABA switch in autistic brain, especially in young children with ASD. Magnetic resonance spectroscopy (MRS) is a promising tool for addressing this question. One study reported a correlation between GABA concentration in the visual cortex (VC) and the degree of perceptual suppression during binocular rivalry in adolescents with typical development, whereas the correlation was significantly lower in autistic patients^20^. Another study reported increased cortical and striatal GABA/glutamate ratios in a neurofibromatosis type 1 mouse model of autism^21^. Hence, the main aim of the current study using MRS is to determine whether bumetanide could regulate GABA/glutamate ratio in the brain and reduce the severity of the autistic symptoms in young children with ASD.

In DSM-5, sensory symptoms are the core diagnostic feature of ASD, which has focused increasing attention on these symptoms. Atypical sensory experience occurs in almost 90% of autistic individuals and affects every sensory modality^22,23^. More broadly, studies have shown that sensory symptoms not only precede but also are predictive of social-communication deficits in childhood, as well as eventual diagnostic status, indicating impaired sensory traits may serve as early biomarkers of autism^24–26^. E-I imbalance has been posited to be the neurobiology of autistic sensory impairment^27,28^. VC is the crucial region involved in visual detection, while MRS studies have been shown to link disruptions in autistic visual processing to GABA concentration in VC ^20,29^. Insular cortex (IC) is well known for its function in sensory integration, which reflects GABA circuit maturation^30^. Impaired multisensory integration was common across four ASD rodent models with autistic behavioural phenotypes and GABAergic circuit abnormalities^30^. Therefore, we chose the VC and IC as the candidate brain regions during MRS scanning, to investigate the effect of bumetanide on “sensory brain”, as well as their relationships with ASD’s core symptoms.

Here, we carried out a pilot open-label trial in which one group of children with ASD aged 3–6 years was administered 1 mg of bumetanide daily for 3 months; matched group of children with ASD that did not receive such treatment served as the control group. We first evaluated the efficacy and safety of bumetanide treatment, then examined the effect on neurotransmitter levels in the brain and the association between the latter and changes in symptoms.

## Materials and methods

### Ethics statement and patients

The observational study was designed to assess the efficacy, safety, and possible neuropharmacological mechanisms of bumetanide in young children with ASD to whom behavioural therapies are not available. The study was conducted in accordance with the provisions of the Declaration of Helsinki and Good Clinical Practice guidelines, and was approved by the Ethics Committee of Xinhua Hospital affiliated to Shanghai Jiao Tong University School of Medicine. The study was registered at the Chinese Clinical Trial Registry (ChiCTR-OPC-16008336). Written informed consent was obtained from the parent or legal guardian of each participant.

The patients, aged 3–6 years old, were recruited at Xinhua Hospital from April 2016 to April 2019 and were diagnosed with ASD according to the Diagnostic and Statistical Manual of Mental Disorders, Fifth Edition. They had no access to behavioural interventions. Diagnosis was confirmed with the Autism Diagnostic Interview–Revised and Autism Diagnostic Observation Schedule (ADOS), and a Children Autism Rating Scale (CARS) total score of no less than 30. The evaluations were performed by a multidisciplinary team that included well-trained specialists, psychologists, and developmental behavioural paediatricians who did not participate in the study. The inclusion of each participant was reviewed by a developmental behavioural paediatrician with over 10 years of professional experience. Exclusion criteria were liver and kidney dysfunction; a history of allergy to sulfa drugs; abnormal electrocardiography; genetic or chromosomal abnormalities; suffering from nervous system diseases (e.g., epilepsy, schizophrenia, etc.). Patients were excluded if they were currently using melatonin for the treatment of sleep disorders or had taken melatonin within the last 3 weeks. This was to eliminate the possibility of melatonin effects on the GABAergic system^31–33^. Additional exclusion criteria for neuroimaging were any contraindications of magnetic resonance imaging (MRI) scanning and any reported structural abnormalities in the brain.

### Experimental design

**Fig. 1:**
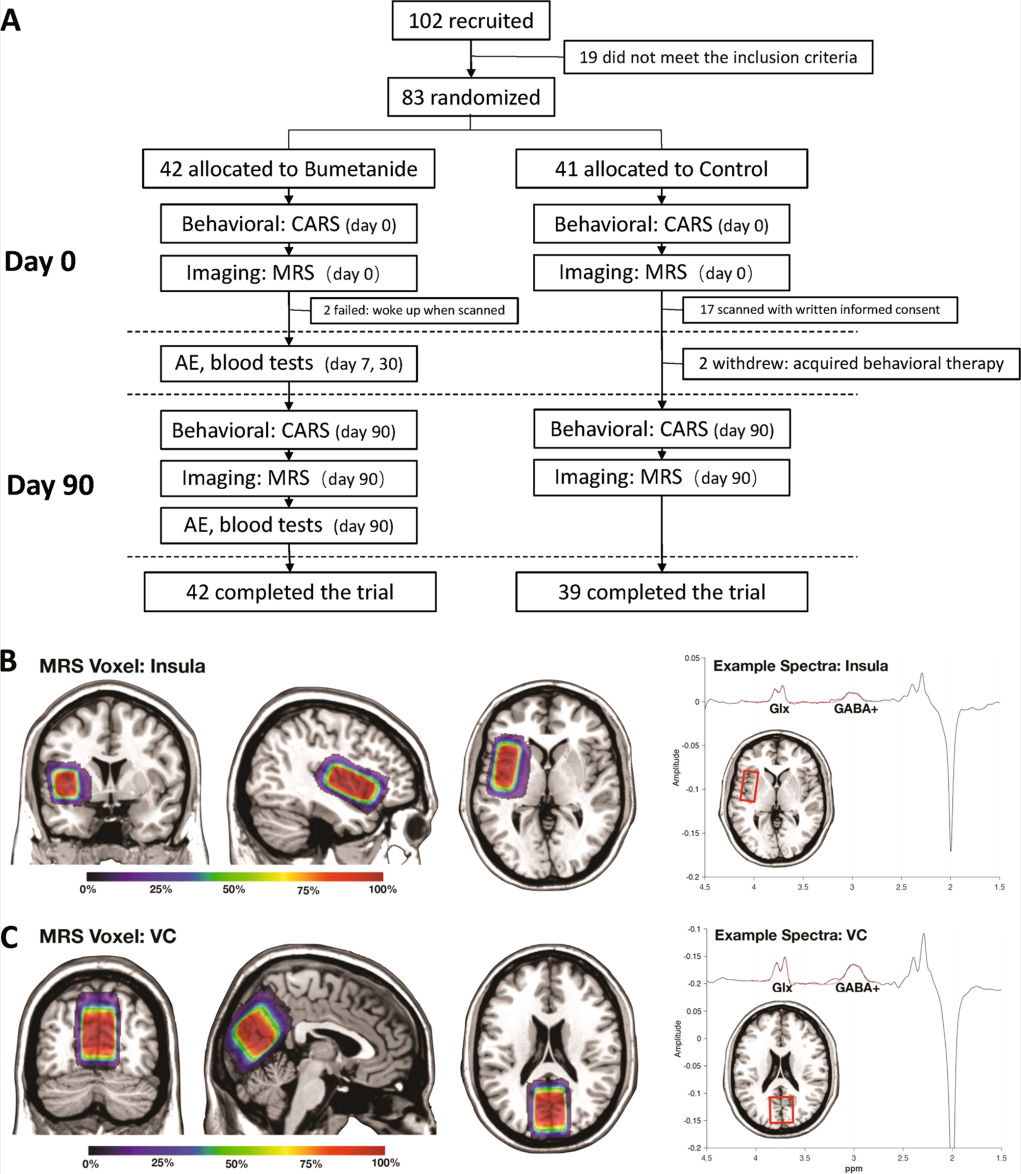
Flow diagram and VOIs in MRS. **a** Among 102 participants recruited in this study, 19 did not meet the inclusion criteria and 83 were randomized into two groups. During the intervention stage, two participants in the control group withdrew, as they were given an opportunity to engage in behavioural therapy. In the end, 81 participants completed this trial; and 57 of them (bumetanide group: *n* = 40, all the parents agreed to scan while two participants waked up during scan; control group: *n* = 17, only 17 parents agreed to scan) were scanned both at baseline and after 3 months. **b**, **c** This figure shows the mean voxel placements in (B left) insula and (C left) visual cortex (VC) from all the studied participants that transformed to the MNI standard space. The colormap indicates the percentage of overlapping. We also show the examples of GABA-edited specta and the fitted GABA/Glx concentrations from (B right) insula and (C right) VC of a representative participant.

The participants were randomly assigned to two groups: bumetanide group, receiving 3-month bumetanide treatment, and control group without such treatment. The trial lasted for 3 months. Behavioural assessment and MRS scanning were performed at baseline and after 3 months. The CARS and other evaluations were conducted ‘blind’ to condition (Bumetanide or no treatment) by experience clinicians. Bumetanide treatment consisted of two 0.5 mg tablets per day for 3 months, given at 8:00 am and 4:00 pm. During the intervention, side effects were closely monitored especially at 1 week and 1 month after the initiation of treatment and at the end of the treatment period. Symptoms (thirst, diuresis, nausea, vomiting, diarrhoea, constipation, rash, palpitation, headache, dizziness, shortness of breath, and any other self-reported symptoms) and blood parameters (serum potassium, uric acid, and creatine) were evaluated. The study procedure is outlined in Fig. 1a.

### Measures

#### Primary outcome

Primary outcome was measured with CARS^34^, which has been used to assess the occurrence and severity of clinical symptoms of ASD and to delineate disease trajectory and monitor treatment efficacy. CARS has 15 items rated on a 7-point scale (1, 1.5, 2, 2.5, 3, 3.5, 4); the total score was calculated by adding the scores of the 15 items; the number of items that assigned a score ≥ 3 was also used to measure the severity of the symptom.

#### Secondary outcome

The Clinical Global Impression (CGI) scale^35^ was used to measure secondary outcome. The CGI-Improvement scale (CGI-I) was used to evaluate the degree of improvement in the patient’s symptoms relative to the baseline, and the CGI-Efficacy Index (CGI-EI) assessed the clinical efficacy of bumetanide by considering both the treatment effect and associated side effects.

#### Exploratory outcomes

Inhibitory (GABA) and excitatory (glutamate [Glx]) neurotransmitter concentrations within a volume of interest (VOI) were measured by MRS. In addition to the VC, we also selected the IC as our VOI, as abnormal GABAergic transmission has been reported in this region in mouse models of autism.

### Imaging

#### Image acquisition

Imaging was performed in the afternoon between 12:00 and 16:00. In our preliminary study, of the 10 children who participated in the scanning session only four slept naturally; two of these subjects awoke after completing the T1 and T2 scans, and none slept through the entire scan. Given the young age and clinical features of our subjects, we offered sedation (50 mg/kg chloral hydrate at a maximum dose of 1 g administered rectally). In a recent review of over 300 children, Karaoui et al have demonstrated the safety and efficacy of chloral hydrate for use in paediatric sedation^36^. Children were scanned 15–20 min after sleep onset, as a comparable stage of slow wave sleep was achieved within this timeframe as during an afternoon nap^37^. The experimental design and the analyses used controlled for any potential confounding effects of chloral hydrate on MRS measures.

Participants were scanned using a Siemens Verio 3.0-Tesla MRI scanner (Siemens Medical Solutions, Munich, Germany) with 32-channel head coil and four-channel neck coil. Earplugs, earphones, and extra foam padding were provided to the subjects to reduce the sound of the scanner during the scan. The insula voxel (20 × 40 × 20 mm, Fig. 1b) was placed along the anterior–posterior direction of the IC and covered the anterior and posterior limits of the insula^38^. The VC voxel (30 × 30 × 30 mm, Fig. 1c) was positioned medially between bilateral occipital cortices^39^. To ensure the consistency of VOI positioning in the longitudinal experiments, we used the first-scanned VOI of each participant as a reference to locate the same VOI in the follow-up scan. For each participant, a three-plane localiser image was first acquired, followed by a high-resolution anatomical T1-weighted magnetisation‐prepared rapid gradient echo image (192 sagittal slices; voxels = 1 × 1 × 1 mm; repetition time [TR] = 2300 ms; echo time [TE] = 2.28 ms; inversion time = 1100 ms; flip angle = 8°, field of view = 192 × 192 × 192 mm) to guide the spectroscopic VOI. For GABA measurements, Mescher-Garwood point-resolved spectroscopy (MEGA-PRESS) scans (256/128 spectra for IC/VC were acquired with on-/off-resonance frequency = 1.9/7.5 ppm using TR/TE = 1500/68 ms) were performed in two VOIs^40^. The difference spectrum was obtained by subtracting the edit-ON and -OFF spectra, yielding a spectrum for total GABA.

#### Imaging preprocessing

For reliable quantification of the GABA signal, we used LCModel software^41,42^ with a simulated MEGA-PRESS basis set to fit the MRS data and determined n-acetylaspartate (NAA), n-acetylaspartyl-Glx, GABA, Glx, glutamine, and glutathione concentrations using the difference spectra. Each spectrum was reviewed and the quality control parameters from LCModel were applied to ensure an acceptable signal-to-noise ratio (SNR) for the MRS voxel. Participants with SNR ≤ 15, full-width at half-maximum ≥ 0.05 ppm, and Cramer–Rao lower bounds in the fitted spectrum equal to or higher than 20% for GABA were excluded from further analysis. Figure 1b, c shows representative MRS spectra of the two target VOIs analyzed with the LCmodel.

GABA metabolite concentrations are expressed in institutional units after normalizing by NAA concentration within the VOI^43^, since there was no inter-group difference either before or after treatment and no significant time × group interaction effect for NAA concentration (Supplementary Table 1). In addition, due to the difference in tissue composition of each VOI (partial volume effect), tissue correction was performed for GABA-edited MRS to adjust GABA measurements. We segmented the T1-weighted images into grey matter (GM), white matter (WM), and cerebrospinal fluid using Statistical Parametric Mapping (SPM12; Wellcome Institute of Neurology, University College London, UK) to compute the volume fraction of each tissue component covered within the VOI^44^. The corrected GABA metabolite concentration was calculated with the following equation: Metabolite_corrected_ = Metabolite_raw_/NAA_observed_ × (1/[fr^GM^ + 0.5 × fr^WM^]).

After quality control, pre- and post-treatment measurements of IC/VC VOIs were available for 52/53 and 46/53 patients, respectively, whereas data at both time points were available for 43/50 patients.

### Statistical analysis

#### Effect on clinical symptoms

Before and after treatment, inter-group differences in demographic parameters (i.e., sex proportion, age, intelligence quotient [IQ]) and symptom severity (i.e., ADOS and CARS) were evaluated with the Welch’s *t* test (*t* statistic, assuming non-equal variances) for continuous variables and Pearson’s chi-squared test for categorical variables.

A mixed-effects model was used to determine whether the group and time × group interaction were significant^45^. Considering sex, age, and IQ as covariates, we tested the fixed effects of time (0, month before treatment; 1, month after treatment), group (0, control; 1, bumetanide), and their interaction (time × group) by assuming different random intercepts for each subject. Our dependent variables were the behavioural assessments (CARS total score and number of scores ≥ 3). The normality of the model residuals was assessed with the Shapiro–Wilk normality test, and homogeneity of variance across groups was evaluated with Levene’s test. If at least one of the two tests were significant, a permutation-based mixed-effects model was established by 3000 random permutations of the group label using the *permlmer* and *predictmeans* functions in R package v.1.0.1 (https://www.r-project.org/). If the interaction term was significant for overall symptoms, we further examined the 15 subscales of the CARS to identify those that were the most affected by the treatment. Using the *p* values from the permutation test (perm.p), we carried out a false discovery rate (FDR) correction for multiple comparisons (fdr.p). For CGI-I and CGI-EI, the Kruskal–Wallis tests were applied to assess the significance level of the inter-group difference.

#### Effect on MRS measurements

For MRS measurements, a linear model with age, sex, and IQ as covariates was used to assess the main effect of group on neurotransmitter concentrations. To directly test the treatment effect on these measurements, we used a mixed-effects model similar to that described above for behavioural assessment. Since the normality tests of the model residuals yielded a few significant results, we combined the permutation-based *p* value of the interaction term in the mixed-effects model with the FDR correction for multiple comparisons among multiple MRS measurements and/or brain regions.

#### Association between changes in MRS measurements and severity of clinical symptoms

We calculated Spearman’s correlation coefficients between the change in MRS measurements after treatment and the change in symptom severity while considering age, sex, IQ, symptom severity, and MRS measurements before treatment as covariates. If a significant association was detected, we further investigated which subscales (i.e., phenotype) of the CARS were associated with the change in MRS measurement based on 3000 random permutations.

To determine whether the baseline MRS measurement reflected the efficacy of bumetanide treatment (i.e., could serve as a predictor of efficacy), the bumetanide group was divided into high and low concentration subgroups based on the median MRS measurement. The Kruskal–Wallis rank sum test was used to assess overall differences among the two bumetanide groups and control group. The FDR was applied to correct for multiple comparisons. If the Kruskal–Wallis test was significant, a post hoc comparison was carried out with Dunn’s test in the Fish Stock Assessment package of R v.0.8.22 software.

All analyses were performed using R v.3.5.1. The code is available from the following webpage: https://github.com/qluo2018/RCT.

## Results

### Demographics and clinical characteristics of the study population

A total of 102 patients were recruited in outpatient settings and 83 met the criteria for study enrolment. Among these patients, 42 received bumetanide treatment (0.5 mg twice daily for 3 months) while 41 control subjects received no such treatment (Fig. 1a). There were no differences between the two groups in terms of symptoms and demographic characteristics before treatment (Table 1). Eighty-one participants completed the trial. Two participants in the control group withdrew, as they were given an opportunity to engage in behavioural therapy. Fifty-seven participants were scanned both at baseline and after 3 months (Fig. 1a).

**Table 1:** Demographic information and clinical characteristics of the study population.

	Control					Bumetanide					Degree of freedom	Statistic	*p* value
	Num.	Min	Max	Mean	SD	Num.	Min	Max	Mean	SD			
Sex (F, M)	12, 29					6, 36					1.00	2.7422	0.098
Age	41	3	6.69	3.97	1.01	42	3.01	6.04	4.19	0.95	80.42	−0.9949	0.3228
IQ	41	35	97	62.28	16.51	42	27.4	90	60.84	13.75	77.74	0.4320	0.6669
ADIR													
TotalA	41	12	30	19.39	4.85	42	11	29	20.40	4.20	79.80	−1.0184	0.3116
TotalBv	34	6	21	13.65	4.39	24	8	23	13.96	3.85	53.30	−0.2860	0.7760
TotalBnv	7	7	14	11.29	2.29	18	5	14	11.11	2.91	13.96	0.1582	0.8766
TotalC	41	3	11	6.07	2.18	42	3	10	5.98	1.99	79.94	0.2111	0.8333
TotalD	41	1	6	3.83	1.69	42	1	6	4.07	1.35	76.50	0.7209	0.4731
ADOS													
TotalA	41	3	10	6.34	1.59	42	3	8	6.21	1.34	77.96	0.3941	0.6946
TotalB	41	7	113	13.37	16.09	42	5	14	10.52	2.35	41.67	1.1196	0.2693
TotalC	41	11	22	17.27	3.38	42	9	22	16.74	3.44	81.00	0.7090	0.4804
TotalD	41	0	6	2.85	1.54	42	0	6	2.64	1.34	78.89	0.6641	0.5086
TotalE	41	0	4	1.95	1.05	42	0	5	2.26	1.21	79.86	−1.2510	0.2146
CARS													
Total score	41	30	46.5	38.15	4.04	42	31.5	43.5	37.40	3.20	76.12	0.9267	0.3570
Num.item ≥ 3	41	1	15	6.32	3.57	42	1	12	5.93	3.03	78.31	0.5341	0.5948

Numbers of subjects with a particular characteristic are listed as integers, and quantitative measurements are presented as mean values ± standard deviations. Inter-group differences are evaluated with Welch’s *t* test for continuous variables and Pearson’s chi-squared test for categorical variables

*IQ* intelligence quotient, *ADIR* The Autism Diagnostic Interview-Revised, *TotalBv* Total B score of ADIR for verbal subjects, *TotalBnv* Total B score for nonverbal subjects, *CARS* Childhood Autism Rating Scale, *Num. item* ≥ 3 number of items assigned a score ≥ 3

### Safety and tolerability of bumetanide treatment

In the bumetanide group, no patient withdrew from the trial due to the adverse effects. The most frequent adverse effect observed was polyuria/pollakiuria (*n* = 15), usually occurred within 3 h after bumetanide administration, which was mild and no additional treatment required. A total of four patients developed mild hypokalemia, with serum potassium between 3.0 and 3.5 mmol/L. These patients were administered potassium supplements and were advised to intake potassium-rich foods. These procedures permitted the return of potassium to normal levels. The remaining adverse effects were loss of appetite (*n* = 4), fatigue (*n* = 1), and mild hyperuricemia (*n* = 1). These results show that bumetanide is safe in young children with ASD when the administration is monitored.

### Bumetanide improves ASD symptoms

The bumetanide and control groups had similar CARS total scores before treatment (Table 1), but after treatment the former had a lower total score (*t*_77.3_ = 3.35, *p* = 0.0012; Cohen’s *d* = 0.74) and less number of items ≥ 3 (*t*_74.6_ = 2.88, *p* = 0.0053; Cohen’s *d* = 0.63). Bumetanide treatment altered the progression of symptoms, as evidenced by significant interaction effects of time × group on CARS total score (*t*_81_ = −9.69, *p* = 3.46 × 10^−15^) and number of scores ≥ 3 (*t*_81_ = −5.31, *p* = 9.38 × 10^−7^) (Table 2 and Supplementary Fig. 1). The normality test of residuals and heteroscedasticity test for the two linear models were non-significant. After correction for multiple comparisons, we found that such interaction effects were particularly significant on six subscales of CARS—namely, item 1 (impairment in human relationships; *t*_81_ = −2.85, fdr.perm.p = 0.02), item 3 (inappropriate affect; *t*_81 = _−3.17, fdr.perm.p = 0.02), item 4 (bizarre use of body movement and persistence of stereotypes; *t*_81_ = −2.83, fdr.perm.p = 0.02), item 5 (peculiarities in relating to non-human objects such as toys and other materials; *t*_81_ = −2.48, fdr.perm.p = 0.046), item 7 (peculiarities of visual responsiveness; *t*_81_ = −2.44, fdr.perm.p = 0.049), and item 13 (activity level; *t*_81_ = −2.83, fdr.perm.p = 0.02)(Table 2).

**Table 2:** Clinical improvement in CARS after bumetanide administration.

CARS score	Control (*n* = 41)				Bumetanide (*n* = 42)				Time × Group					
	Min	Max	Mean	SD	Min	Max	Mean	SD	*t*_81_	*p* value	Shapi.p	Leven.p	perm.p	fdr.p
Total score	30	47	37.27	4.09	27.5	40.5	34.51	3.35	−9.69	3.46 × 10^−15^	0.9149	0.3301		
Num.item ≥ 3	0	15	5.49	3.50	0	9	3.52	2.65	−5.31	9.38 × 10^−7^	0.9059	0.0846		
Item 1	1.5	3.5	2.71	0.46	1.5	4	2.50	0.53	−2.85	0.0055	0.0019	0.7963	0.0053	0.0200
Item 2	1.5	3.5	2.37	0.54	2	4	2.37	0.43	−2.18	0.0319	0.0015	0.2847	0.0350	0.0750
Item 3	2	3.5	2.65	0.39	2	3	2.40	0.43	−3.17	0.0022	0.5326	0.4607	0.0017	0.0200
Item 4	1	3	2.50	0.50	1	3.5	2.30	0.55	−2.83	0.0058	0.0018	0.8898	0.0033	0.0200
Item 5	2	3.5	2.66	0.48	1.5	3	2.43	0.44	−2.48	0.0151	0.1594	0.3898	0.0153	0.0460
Item 6	1.5	3.5	2.37	0.49	1	3	2.08	0.40	−1.71	0.0906	1.85 × 10^−6^	0.0164	0.0923	0.1538
Item 7	2	3	2.55	0.44	1	3	2.30	0.43	−2.44	0.0170	2.92 × 10^−6^	0.2265	0.0197	0.0492
Item 8	1.5	3	2.40	0.44	1	3	2.25	0.47	−1.90	0.0615	0.0993	0.8441	0.0606	0.1137
Item 9	1	3.5	2.20	0.61	1	3	1.79	0.54	−0.76	0.4517	4.63 × 10^−9^	0.4441	0.4285	0.4945
Item 10	1	3	2.05	0.43	1	2.5	1.58	0.49	−1.36	0.1771	4.21 × 10^−5^	0.1515	0.1716	0.2340
Item 11	1.5	3	2.71	0.49	1.5	4	2.79	0.47	−1.25	0.2156	6.18 × 10^−5^	0.4268	0.2166	0.2707
Item 12	1	3	2.28	0.61	1.5	3	2.25	0.47	−1.45	0.1501	0.0023	0.0828	0.1466	0.2199
Item 13	1.5	3	2.59	0.46	1.5	3.5	2.30	0.44	−2.83	0.0059	7.46 × 10^−6^	0.2050	0.0050	0.0200
Item 14	2	4	2.38	0.47	2	3	2.27	0.44	−0.43	0.6676	1.90 × 10^−24^	0.3272	0.6768	0.7251
Item 15	1.5	3.5	2.88	0.56	2	4	2.98	0.61	−0.26	0.7950	5.11 × 10^−17^	0.5057	0.7611	0.7611

*CARS* Childhood Autism Rating Scale, *Num. item* ≥ 3 number of items assigned a score ≥ 3, *Shapi.p* *p* value of the Shapiro–Wilk normality test for model residuals, *Leven. p* *p* value of Levene’s test for homogeneity of variance, *perm.p* *p* value for time × group interaction in a mixed effect model based on 3000 random permutations, *fdr.p* false discovery rate corrected *p* value

Using CGI, clinical improvement was confirmed by both the improvement scale (CGI-I; kw-*χ*^2^ = 17.09, *p* = 3.56 × 10^−5^) and the efficacy index (CGI-EI; kw-*χ*^2^ = 11.89, *p* = 5.62 × 10^−4^).

### Bumetanide alters GABA/NAA and GABA/Glx ratios in the IC

**Fig. 2:**
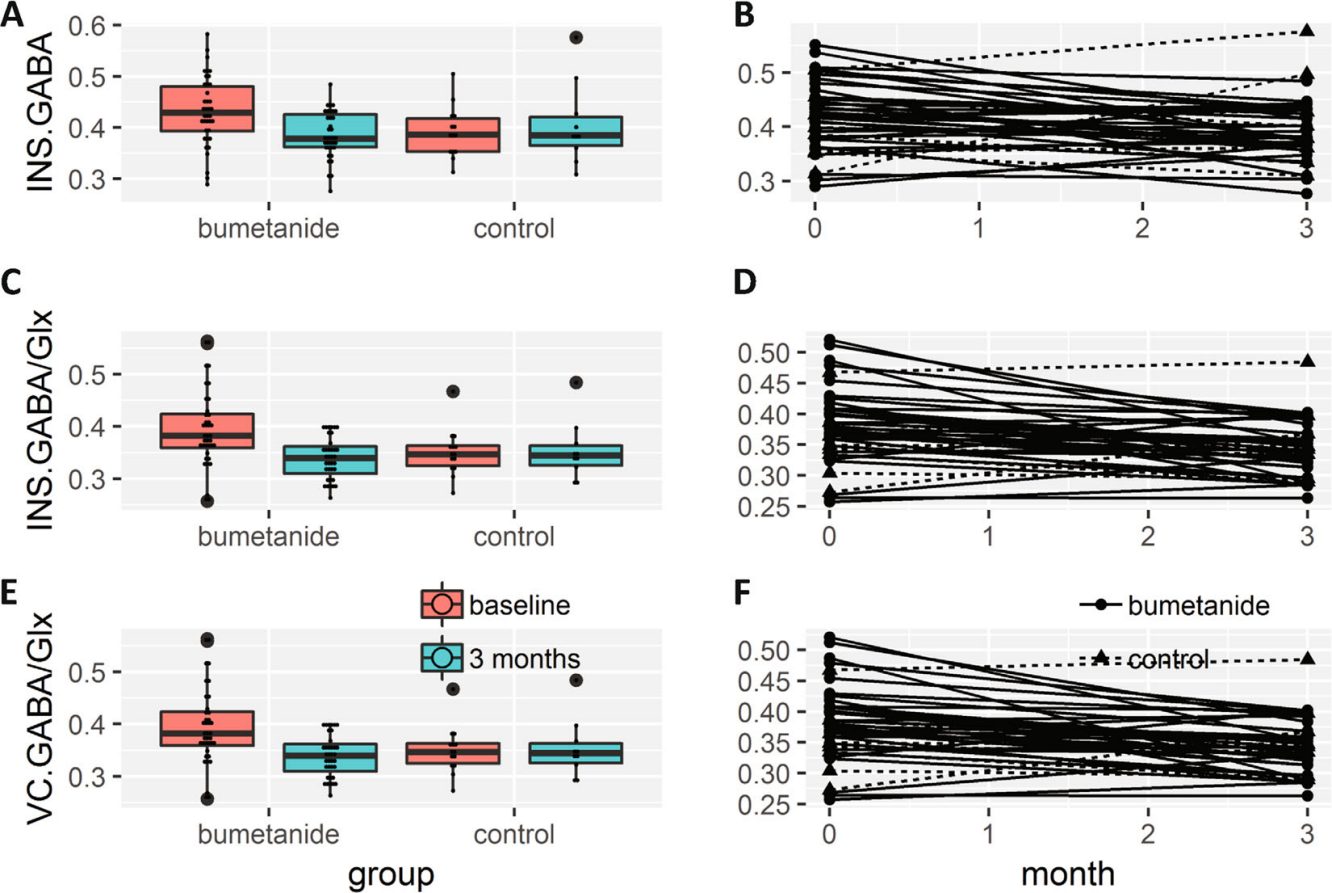
Changes in neurotransmitter levels after bumetanide administration. **a** Group comparisons of insular GABA concentration before and after treatment. Dots represent insular GABA concentration of each subject. **b** Trajectories of the change of insular GABA concentration before and after treatment. Dots represent GABA concentration of each subject from bumetanide group, and triangles represent the concentration of control. **c**, **e** Group comparisons of insular GABA/Glx ratios (**c**) visual GABA/Glx ratios (**e**) before and after treatment. **d**, **f** Trajectories of the change of both the insular GABA/Glx ratios (**d**) and the visual GABA/Glx ratios (**f**). Dots represent GABA/Glx ratios of each subject from bumetanide group, and triangles represent the concentration of control. Abbreviations: INS-insular cortex, VC-visual cortex, GABA -γ-aminobutyric acid, Glx – glutamate and glutamine.

Before treatment, the bumetanide group had a higher GABA/NAA ratio (*F*_1,47_ = 5.27, perm.p = 0.0260) in the IC and a higher GABA/Glx ratio in both the IC (*F*_1,47_ = 4.80, perm.p = 0.0463) and VC (*F*_1,48_ = 11.01, perm.p = 0.0013) compared with the control group (Fig. 2 and Table 3). Consistent with the trends in the symptoms, we found that bumetanide had significant effects over the 3-month treatment course on both GABA/NAA (*F*_1,41_ = 5.06, fdr.perm.p = 0.0418) and GABA/Glx (*F*_1,41_ = 6.16, fdr.perm.p = 0.0418) ratios in the IC and GABA/Glx ratio (*F*_1,48_ = 5.47, fdr.perm.p = 0.0418) in the VC when considering baseline MRS measurements as additional covariates (Fig. 2 and Table 3).

**Table 3:** Magnetic resonance spectroscopy changes after bumetanide administration.

MRS measures		Control			Bumetanide			Group comparisons				
		*n*	Mean	SD	*n*	Mean	SD	*F*	f.p	f.df	perm.p	fdr.p
Group (before treatment)	INS.GABA/NAA	14	0.39	0.05	38	0.43	0.07	5.27	0.0262	47	0.0260	
	INS.GABA/Glx	14	0.35	0.05	38	0.39	0.07	4.80	0.0335	47	0.0463	
	VC.GABA/NAA	17	0.38	0.06	36	0.40	0.05	2.73	0.1049	48	0.1550	
	VC.GABA/Glx	17	0.40	0.06	36	0.46	0.06	11.01	0.0017	48	0.0013	
Group (after treatment)	INS.GABA/NAA	10	0.41	0.08	36	0.39	0.05	1.20	0.2801	41	1.0000	
	INS.GABA/Glx	10	0.35	0.06	36	0.34	0.04	0.83	0.3673	41	0.9983	
	VC.GABA/NAA	15	0.36	0.05	38	0.37	0.05	0.04	0.8502	48	0.9280	
	VC.GABA/Glx	15	0.42	0.05	38	0.42	0.05	0.06	0.8037	48	0.9427	
Time × Group (change between before and after treatment)	INS.GABA/NAA							5.06	0.0299	41	0.0313	0.0418
	INS.GABA/Glx							6.16	0.0173	41	0.0147	0.0418
	VC.GABA/NAA							1.41	0.2406	48	0.2279	0.2279
	VC.GABA/Glx							5.47	0.0236	48	0.0293	0.0418

*perm.p* *p* value given by 3000 random permutations, *fdr.p* false discovery rate corrected *p* value

### Changes in the GABA/Glx ratio in the IC are associated with clinical improvement

The change in GABA/Glx ratio in the IC was associated with a decrease in the number of CARS scores ≥ 3 in the bumetanide group (Spearman’s *r* = 0.42, *p* = 0.0194, *n* = 35); this correlation was stronger in the bumetanide group than in the control group (*z* = 5.71, *p* = 0.0347). We also found both significant association with CARS item4 (bizarre use of body movement and persistence of stereotypes) (*r* = 0.37, *p* = 0.0466, *n* = 35) and item 5 (peculiarities in relating to non-human objects such as toys and other materials) (*r* = 0.47, *p* = 0.0084, *n* = 35) (Supplementary Table 2).

Using the median GABA/Glx ratio in the IC (0.3691) as a threshold, we tested whether such a pre-treatment ratio could predict treatment efficacy. Non-parametric inter-group comparisons revealed differences in CGI-I (kw-*χ*^2^ = 13.64, *p* = 0.0011; Supplementary Table 3). The post hoc tests indicated that the bumetanide subgroup with a sub-threshold GABA/Glx ratio showed greater improvement than the bumetanide subgroup with a higher GABA/Glx ratio (kw-*χ*^2^ = 1.99, fdr.p = 0.0468) and the control group (kw-*χ*^2^ = −3.69, fdr.p = 0.0007), with the latter two groups showing comparable change (fdr.p = 0.0532) (Supplementary Table 3).

## Discussion

The present study showed that treatment with bumetanide reduced the severity of ASD symptoms in a group of patients, and also demonstrated the safety of this drug for children aged between 3 and 6 years. Importantly, our results provide the first neuroimaging evidence that the GABA/Glx ratio was decreased in both the IC and VC relative to the control group following bumetanide administration. Furthermore, the decrease in GABA/Glx ratio was associated with a reduction in symptom severity as assessed by the CARS. These findings provide insight into the mechanistic basis for the clinical efficacy of bumetanide in ASD and support to the hypothesis that bumetanide can restore E-I balance in the autistic brain thereby promoting normal brain function and social emotional cognition.

It is thought that GABAergic signals are altered in ASD and that an elevated level of [Cl^−^]_i_ contributes to E-I imbalance during early brain development^46^. In mouse models of ASD, bumetanide was shown to function as a high-affinity specific inhibitor of NKCC1 that could reduce [Cl^−^]_i_ and thus restore GABAergic transmission to rescue autism-like behaviours^11–14^. However, direct neuropharmacological evidence for the action of bumetanide in humans is lacking, despite the promising therapeutic effects in ASD patients reported in several clinical studies. Using ^1^H MRS MEGA-PRESS, a state-of-the-art neuroimaging sequence, we detected both GABA and Glx in cortical neurons in vivo, which allowed us to monitor E-I transmission in target VOIs during brain development.

The observed association between the decreased GABA/Glx ratio in the IC and improvement in CARS score after bumetanide treatment provides evidence for the involvement of this cortex in ASD. The insula has traditionally been recognized as a limbic integrative cortex that is part of a so-called salience network, which integrates sensory, emotional, and autonomic signals in order to allocate cognitive resources and guide behaviour^47^. In particular, impaired sensory integration and the underlying GABAergic circuits in the IC were rescued in mouse models of ASD by pharmacological enhancement of inhibitory transmission^30^. Neuroimaging evidence has revealed hypoactivity of the insula in ASD patients in response to social stimuli^48^. Thus, the most likely explanation for our results is that bumetanide decreased the severity of ASD symptoms by improving sensory integration and decreasing salience of non-social stimuli while enhancing salience of social emotion stimuli^49^. This was supported by the observed association between the decrease in GABA/Glx ratio in the IC and improvement of the symptoms “bizarre use of body movement and persistence of stereotypes” and “peculiarities in relating to non-human objects (like toys and other materials)” following bumetanide administration.

The measurement of GABA/Glx ratio in the insula by MRS is a promising tool for the precision medicine approach to ASD treatment^50^. The lower pre-treatment GABA/Glx ratio in the IC was associated with greater clinical improvement at the 3-month follow-up. Interestingly, before the initiation of bumetanide therapy there was no difference in symptom severity between patients with higher and lower GABA/Glx ratios. Thus, patients with similar symptom scores can present distinct neuroimaging findings; moreover, quantitative indices such as GABA/Glx ratio in the insula are potential biomarkers for monitoring treatment response.

Indeed, the precise mechanistic links between bumetanide-mediated reducing intracellular chloride concentration([Cl^−^]_i_) and the resultant decrease of GABA/glutamate ratios in ASD patients warrant further investigation. In the literature, it was reported that the GABA switch (excitatory-to-inhibitory GABA switch) was impaired in ASD, and bumetanide was able to largely correct this impairment via gradually restoring the low level of [Cl^−^]_i_ and autistic behaviours in ASD rodent models^11,51^. In our present work, it is not feasible yet to test whether bumetanide restores the impaired GABA switch in ASD patients. Mechanistically, we inferred the bumetanide-caused decrease in GABA might be considered as a reduction in an excitatory action of GABA, followed by the shift of polarity of GABA and then a gradually enhanced GABAergic inhibition. In the future, prolonged monitoring of GABA and GABA/Glx would be helpful to illustrate this dynamic change of these neurotransmitters during the GABA switch associated with the neurodevelopment and bumetanide treatment. In summary, although the precise mechanisms underlying the effects of bumetanide on the GABA concentration in insular and VCs remain to be established, we favoured the notion that bumetanide could restore the imbalance between neuronal excitation and inhibition in ASD, and the current shift in GABA/glutamate levels might reflect the process of the remodelling of GABAergic inhibition.

Kaila et al. have questioned the brain availability of bumetanide due to its low permeability of brain-blood barrier (BBB) and rapid efflux^52^. Indeed, in neurodevelopmental disorders, the BBB has been suggested to be especially permissive^53,54^. Our study in young children supported the previous reports that bumetanide can alleviate the core symptoms of ASD, and importantly advances the understanding of the mechanism in the brain by which these effects occurred. A recent review^55^ has indicated that the mechanisms controlling bumetanide’s brain entry and removal are complex. New transport mechanisms have been identified recently and it is conceivable that the pharmacokinetics of bumetanide or the affinity of phosphorylated NKCC1 are modified in developmental disorders.

There were several limitations to this study. First of all, as a pilot study, the small sample size prevented more detailed profiling of the best responders to this treatment and establishment of the optimal time window for intervention. Additionally, while the MRS measurements weren’t balanced between the two groups at baseline, we controlled for the potential limitation by covariate analyses. In future, large-scale, multi-center studies will be able to address this limitation. Nevertheless, these are novel and highly clinically relevant findings, which provide a mechanism for the improvement in ASD patients by bumetanide. Moreover, brain regions involved in sensory information processing/integration were investigated in this study. In future MRS research focused on the social-communication and cognitive-associated brain regions, such as ventromedial prefrontal cortex, are needed to further elucidate the neuropharmacological mechanism of bumetanide. Last but not the least, although the results of our study are promising, a double-blind, randomised clinical trial with a larger population is required to confirm the efficacy of bumetanide treatment for ASD.

## Conclusions

In summary, the results of this study demonstrate that bumetanide has clinical potential for the treatment of ASD, with few side effects; and that the GABA/Glx ratio in the IC is a useful neuroimaging biomarker for monitoring the response to treatment.

## Supplementary information

Supplementary Methods

Supplementary Table 1

Supplementary Table 2

Supplementary Table 3

Supplementary Figure 1

## References

[1] Baio J (2018). Prevalence of autism spectrum disorder among children aged 8 years - autism and developmental disabilities monitoring network, 11 sites, United States, 2014. Morbidity Mortal. Wkly. Rep. Surveill. Summaries.

[2] Sun X (2019). Autism prevalence in China is comparable to Western prevalence. Mol. Autism.

[3] Pierce K. et al. Evaluation of the diagnostic stability of the early autism spectrum disorder phenotype in the general population starting at 12 months. *JAMA Pediatr*. **173**, 578–587 (2019). 10.1001/jamapediatrics.2019.0624 PMC6547081 31034004

[4] Inui T, Kumagaya S, Myowa-Yamakoshi M (2017). Neurodevelopmental hypothesis about the etiology of autism spectrum disorders. Front Hum. Neurosci..

[5] Lord C, Elsabbagh M, Baird G, Veenstra-Vanderweele J (2018). Autism spectrum disorder. Lancet.

[6] Salomone E (2016). Use of early intervention for young children with autism spectrum disorder across Europe. Autism Int. J. Res. Pract..

[7] Hastings RP, Robertson J, Yasamy MT (2012). Interventions for children with pervasive developmental disorders in low and middle income countries. J. Appl. Res. Intellect. Disabil..

[8] Represa A, Ben-Ari Y (2005). Trophic actions of GABA on neuronal development. Trends Neurosci..

[9] Rivera C (1999). The K+/Cl− co-transporter KCC2 renders GABA hyperpolarizing during neuronal maturation. Nature.

[10] Lemonnier E (2017). Effects of bumetanide on neurobehavioral function in children and adolescents with autism spectrum disorders. Transl. Psychiatry.

[11] Tyzio R (2014). Oxytocin-mediated GABA inhibition during delivery attenuates autism pathogenesis in rodent offspring. Science.

[12] Roux S, Lohof A, Ben-Ari Y, Poulain B, Bossu JL (2018). Maturation of GABAergic transmission in cerebellar Purkinje cells is sex dependent and altered in the valproate model of autism. Front. Cell. Neurosci..

[13] Fernandez A. et al. The GABA developmental shift is abolished by maternal immune activation already at birth. *Cereb. Cortex* **29**, 3982–3992 (2019). 10.1093/cercor/bhy279 30395185

[14] Lozovaya N (2019). Early alterations in a mouse model of Rett syndrome: the GABA developmental shift is abolished at birth. Sci. Rep..

[15] Lemonnier E, Ben-Ari Y (2010). The diuretic bumetanide decreases autistic behaviour in five infants treated during 3 months with no side effects. Acta Paediatrica.

[16] Lemonnier E (2012). A randomised controlled trial of bumetanide in the treatment of autism in children. Transl. psychiatry.

[17] Du L (2015). A pilot study on the combination of applied behavior analysis and bumetanide treatment for children with autism. J. Child Adolesc. Psychopharmacol..

[18] Bruining H (2015). Paradoxical benzodiazepine response: a rationale for bumetanide in neurodevelopmental disorders?. Pediatrics.

[19] Hadjikhani N (2018). Bumetanide for autism: more eye contact, less amygdala activation. Sci. Rep..

[20] Robertson CE, Ratai EM, Kanwisher N (2016). Reduced GABAergic action in the autistic brain. Curr. Biol..

[21] Goncalves J (2017). Testing the excitation/inhibition imbalance hypothesis in a mouse model of the autism spectrum disorder: in vivo neurospectroscopy and molecular evidence for regional phenotypes. Mol. Autism.

[22] Tomchek SD, Dunn W (2007). Sensory processing in children with and without autism: a comparative study using the short sensory profile. Am. J. Occup. Ther..

[23] Tavassoli T, Miller LJ, Schoen SA, Nielsen DM, Baron-Cohen S (2014). Sensory over-responsivity in adults with autism spectrum conditions. Autism.

[24] Estes A (2015). Behavioral, cognitive, and adaptive development in infants with autism spectrum disorder in the first 2 years of life. J. Neurodev. Disord..

[25] Turner-Brown LM, Baranek GT, Reznick JS, Watson LR, Crais ER (2013). The first year inventory: a longitudinal follow-up of 12-month-old to 3-year-old children. Autism.: Int. J. Res. Pract..

[26] Boyd BA (2010). Sensory features and repetitive behaviors in children with autism and developmental delays. Autism Res..

[27] Orefice LL (2016). Peripheral mechanosensory neuron dysfunction underlies tactile and behavioral deficits in mouse models of ASDs. Cell.

[28] Robertson CE, Baron-Cohen S (2017). Sensory perception in autism. Nat. Rev. Neurosci..

[29] Hensch TK (2005). Critical period plasticity in local cortical circuits. Nat. Rev. Neurosci..

[30] Gogolla N, Takesian AE, Feng G, Fagiolini M, Hensch TK (2014). Sensory integration in mouse insular cortex reflects GABA circuit maturation. Neuron.

[31] Acuna-Castroviejo D (2014). Extrapineal melatonin: sources, regulation, and potential functions. Cell. Mol. life Sci..

[32] Marquez de Prado B (2000). Melatonin disrupts circadian rhythms of glutamate and GABA in the neostriatum of the aware rat: a microdialysis study. J. Pineal Res..

[33] Acuna Castroviejo D, Rosenstein RE, Romeo HE, Cardinali DP (1986). Changes in gamma-aminobutyric acid high affinity binding to cerebral cortex membranes after pinealectomy or melatonin administration to rats. Neuroendocrinology.

[34] Schopler E, Reichler RJ, DeVellis RF, Daly K (1980). Toward objective classification of childhood autism: Childhood Autism Rating Scale (CARS). J. Autism Dev. Disord..

[35] Wu, W. Y. Clinical global impression (CGI). *General Psychiatry* **2**, 76–77 (1984).

[36] Karaoui M (2018). Chloral hydrate administered by a dedicated sedation service can be used safely and effectively for pediatric ophthalmic examination. Am. J. Ophthalmol..

[37] Kurth S (2016). Development of nap neurophysiology: preliminary insights into sleep regulation in early childhood. J. sleep. Res..

[38] Wiebking C (2014). GABA in the insula - a predictor of the neural response to interoceptive awareness. NeuroImage.

[39] Gaetz W (2014). GABA estimation in the brains of children on the autism spectrum: measurement precision and regional cortical variation. NeuroImage.

[40] Mescher M, Merkle H, Kirsch J, Garwood M, Gruetter R (1998). Simultaneous in vivo spectral editing and water suppression. NMR Biomed..

[41] Provencher SW (1993). Estimation of metabolite concentrations from localized in vivo proton NMR spectra. Magn. Reson. Med..

[42] Provencher SW (2001). Automatic quantitation of localized in vivo 1H spectra with LCModel. NMR biomedicine.

[43] Stagg CJ, Bachtiar V, Johansen-Berg H (2011). The role of GABA in human motor learning. Curr. Biol..

[44] Harris AD, Puts NA, Edden RA (2015). Tissue correction for GABA-edited MRS: Considerations of voxel composition, tissue segmentation, and tissue relaxations. J. Magn. Reson Imaging.

[45] Nieuwenhuis S, Forstmann BU, Wagenmakers E-J (2011). Erroneous analyses of interactions in neuroscience: a problem of significance. Nat. Neurosci..

[46] Nardou R (2011). Neuronal chloride accumulation and excitatory GABA underlie aggravation of neonatal epileptiform activities by phenobarbital. Brain.

[47] Uddin LQ (2015). Salience processing and insular cortical function and dysfunction. Nat. Rev. Neurosci..

[48] Nomi JS, Molnar-Szakacs I, Uddin LQ (2019). Insular function in autism: update and future directions in neuroimaging and interventions. Prog. Neuro-Psychopharmacol. Biol. psychiatry.

[49] Hadjikhani N (2015). Improving emotional face perception in autism with diuretic bumetanide: a proof-of-concept behavioral and functional brain imaging pilot study. Autism.

[50] Loth E, Murphy DG, Spooren W (2016). Defining precision medicine approaches to autism spectrum disorders: concepts and challenges. Front. Psychiatry.

[51] Cloarec R (2019). Pyramidal neuron growth and increased hippocampal volume during labor and birth in autism. Sci. Adv..

[52] Puskarjov M, Kahle KT, Ruusuvuori E, Kaila K (2014). Pharmacotherapeutic targeting of cation-chloride cotransporters in neonatal seizures. Epilepsia.

[53] Fiorentino M (2016). Blood-brain barrier and intestinal epithelial barrier alterations in autism spectrum disorders. Mol. Autism.

[54] Xhima K, Weber-Adrian D, Silburt J (2016). Glutamate induces blood-brain barrier permeability through activation of N-methyl-D-aspartate receptors. The. J. Neurosci..

[55] Ben-Ari Y (2017). NKCC1 chloride importer antagonists attenuate many neurological and psychiatric disorders. Trends Neurosci..

