# Correction: Symptom improvement in children with autism spectrum disorder following bumetanide administration is associated with decreased GABA/glutamate ratios

**DOI:** 10.1038/s41398-020-0747-4

**Published:** 2020-02-12

**Authors:** Lingli Zhang, Chu-Chung Huang, Yuan Dai, Qiang Luo, Yiting Ji, Kai Wang, Shining Deng, Juehua Yu, Mingyu Xu, Xiujuan Du, Yun Tang, Chun Shen, Jianfeng Feng, Barbara J Sahakian, Ching-Po Lin, Fei Li

**Affiliations:** 1 Developmental and Behavioral Pediatric Department & Child Primary Care Department, Brain and Behavioral Research Unit of Shanghai Institute for Pediatric Research and MOE- Shanghai Key Laboratory for Children’s Environmental Health, Xinhua Hospital, Shanghai Jiao Tong University School of Medicine, Shanghai, China; 2 Shanghai Institute of Pediatric Research, Xinhua Hospital, Shanghai Jiao Tong University School of Medicine, Shanghai, China; 3 Institute of Science and Technology for Brain-Inspired Intelligence, MOE-Key Laboratory of Computational Neuroscience and Brain-Inspired Intelligence, Fudan University, Shanghai, China; 4 State Key Laboratory of Medical Neurobiology and MOE Frontiers Center for Brain Science, Institute of Brain Science and Human Phenom Institute, Fudan University, Shanghai, China; 5 Departments of Psychology and Psychiatry and the Behavioural and Clinical Neuroscience Institute, University of Cambridge, Cambridge, UK; 6 Institute of Neuroscience, National Yang-Ming University, Taipei, Taiwan

**Keywords:** Predictive markers, Autism spectrum disorders

**Correction to**: **Translational Psychiatry**

10.1038/s41398-020-0692-2 published online 27 January 2020

An important detail was omitted in the Method of the original Article, I.E, The CARS and other evaluations were conducted ‘blind' to condition (Bumetanide or no treatment) by experienced clinicians. This has now been updated in the HTML and PDF versions of this Article.

