# Supplementary material for: Symptom improvement in children with autism spectrum disorder following bumetanide administration is associated with decreased GABA/glutamate ratios
Source: Transl Psychiatry. 2020 Jan 27;10:9. doi: 10.1038/s41398-020-0692-2 (PMC7026137; doi:10.1038/s41398-020-0692-2)
Supplement: Supplementary file 1 — Supplementary Methods [file 41398_2020_692_MOESM1_ESM.docx]

**Supplementary Methods**

**Power calculation of the sample size**

Setting Test family as t tests, Statistical test as Means: Difference between two independent means (two groups), Type of power analysis: A priori: Compute required sample size - given α, power, and effect size in G*Power, we estimated the required sample size based on a previous bumetanide study^1^, which showed the symptom improvement assessed by CARS total score was 5.6±4 points in bumetanide group and 1.8±5.1 in placebo group, after 3-month intervention. We estimated the minimum sample size as 78 (39 in each group) for 95% power, assuming a 5% significance level with two-side test. In this study, 83 participants were recruited.

**Strategies to control the confounding effects of chloral hydrate on MRS measurements**

The potential confounding effects of chloral hydrate were controlled for both by experimental design and analyses used. In the experimental design, both the children in the control and the drug group received chloral hydrate. Therefore, the chloral hydrate was administered at both time points to both groups. In addition, in the analysis of the data, we used a mixed effect model instead of a repeated measures ANOVA. By using this approach, we were able to subtract out the “time-invariant” effect of chloral hydrate.

**Reference**

1. Lemonnier E, *et al*. A randomised controlled trial of bumetanide in the treatment of autism in children. *Translational psychiatry* 2012; **2:** e202.
