# Supplementary material for: Symptom improvement in children with autism spectrum disorder following bumetanide administration is associated with decreased GABA/glutamate ratios
Source: Transl Psychiatry. 2020 Jan 27;10:9. doi: 10.1038/s41398-020-0692-2 (PMC7026137; doi:10.1038/s41398-020-0692-2)
Supplement: Supplementary file 2 — Supplementary Table 1 [file 41398_2020_692_MOESM2_ESM.docx]

**Supplementary Table 1. Group comparison of NAA at two brain regions between the bumetanide group and the control group.**

| **MRS measures** | | **control** | | | **bumetanide** | | | **group comparison** | | | | | |
| --- | --- | --- | --- | --- | --- | --- | --- | --- | --- | --- | --- | --- | --- |
|  |  | **n** | **mean** | **sd** | **n** | **mean** | **sd** | **shapi.p** | **levene.p** | **F** | **F.p** | **F.df** | **perm.p** |
| **Baseline** | INS.NAA.tissue.corrected | 14 | 0.0483 | 0.0027 | 38 | 0.0478 | 0.0040 | 0.8924 | 0.1526 | 0.2545 | 0.6163 | 47 | 0.4513 |
|  | VC.NAA.tissue.corrected | 17 | 0.0669 | 0.0088 | 36 | 0.0669 | 0.0057 | 0.4968 | 0.1427 | 0.0000 | 0.9972 | 48 | 0.8163 |
| **Follow-up** | INS.NAA.tissue.corrected | 10 | 0.0479 | 0.0037 | 36 | 0.0480 | 0.0049 | 0.0900 | 0.5930 | 0.0018 | 0.9662 | 41 | 0.7527 |
|  | VC.NAA.tissue.corrected | 15 | 0.0677 | 0.0075 | 38 | 0.0694 | 0.0089 | 0.2019 | 0.6033 | 0.4558 | 0.5028 | 48 | 0.6950 |
| **Time***  **Group** | INS.NAA.tissue.corrected |  |  |  |  |  |  | 0.2034 | 0.3796 | 0.0026 | 0.9597 | 41 | 0.8411 |
|  | VC.NAA.tissue.corrected |  |  |  |  |  |  | 0.0641 | 0.5710 | 0.5868 | 0.4474 | 48 | 0.4389 |
