# Supplementary material for: Symptom improvement in children with autism spectrum disorder following bumetanide administration is associated with decreased GABA/glutamate ratios
Source: Transl Psychiatry. 2020 Jan 27;10:9. doi: 10.1038/s41398-020-0692-2 (PMC7026137; doi:10.1038/s41398-020-0692-2)
Supplement: Supplementary file 3 — Supplementary Table 2 [file 41398_2020_692_MOESM3_ESM.docx]

**Supplementary Table 2. Association between the changes of MRS and the changes of symptoms after the treatment.**

| **Change of MRS** | **Change of symptoms** | **Bumetanide group** | | | | **Control group** | | |
| --- | --- | --- | --- | --- | --- | --- | --- | --- |
|  |  | **n** | **r** | **r.p** | **perm.p** | **n** | **r** | **r.p** |
| **Insular GABA/NAA** | CARS total score | 35 | 0.28 | 0.1282 |  | 8 | -0.79 | 0.4175 |
|  | CARS num.item≥3 | 35 | 0.21 | 0.2633 |  | 8 | -1.00 | 0.0542 |
| **Insular GABA/Glx** | CARS total score | 35 | 0.28 | 0.1388 | 0.2410 | 8 | -0.68 | 0.5263 |
|  | CARS num.item≥3 | 35 | 0.42 | 0.0194 | 0.0347 | 8 | -0.98 | 0.1365 |
|  | CARS item 1 | 35 | 0.03 | 0.8625 | 1.0000 | 8 | 0.84 | 0.3658 |
|  | CARS item 2 | 35 | -0.05 | 0.7798 | 1.0000 | 8 | -0.80 | 0.4063 |
|  | CARS item 3 | 35 | -0.32 | 0.0857 | 0.6957 | 8 | -0.98 | 0.1276 |
|  | CARS item 4 | 35 | 0.37 | 0.0466 | 0.4793 | 8 | -0.86 | 0.3453 |
|  | CARS item 5 | 35 | 0.47 | 0.0084 | 0.1097 | 8 | / | / |
|  | CARS item 6 | 35 | -0.04 | 0.8183 | 1.0000 | 8 | 0.72 | 0.4891 |
|  | CARS item 7 | 35 | 0.07 | 0.7176 | 1.0000 | 8 | 1.00 | 0.0145 |
|  | CARS item 8 | 35 | 0.21 | 0.2696 | 0.9887 | 8 | -0.78 | 0.4314 |
|  | CARS item 9 | 35 | -0.11 | 0.5502 | 1.0000 | 8 | -0.94 | 0.2241 |
|  | CARS item 10 | 35 | -0.35 | 0.0577 | 0.5560 | 8 | 0.82 | 0.3839 |
|  | CARS item 11 | 35 | 0.15 | 0.4206 | 1.0000 | 8 | -0.87 | 0.3280 |
|  | CARS item 12 | 35 | 0.10 | 0.6065 | 1.0000 | 8 | 0.50 | 0.6654 |
|  | CARS item 13 | 35 | -0.01 | 0.9499 | 1.0000 | 8 | 1.86 | / |
|  | CARS item 14 | 35 | / | / | / | 8 | / | / |
|  | CARS item 15 | 35 | 0.32 | 0.0864 | 0.6990 | 8 | -0.97 | 0.1513 |
| **Visual GABA/Glx** | CARS total score | 35 | 0.01 | 0.9776 |  | 15 | 0.30 | 0.3996 |
|  | CARS num.item≥3 | 35 | 0.01 | 0.9389 |  | 15 | 0.31 | 0.3915 |
