# Supplementary material for: Symptom improvement in children with autism spectrum disorder following bumetanide administration is associated with decreased GABA/glutamate ratios
Source: Transl Psychiatry. 2020 Jan 27;10:9. doi: 10.1038/s41398-020-0692-2 (PMC7026137; doi:10.1038/s41398-020-0692-2)
Supplement: Supplementary file 4 — Supplementary Table 3 [file 41398_2020_692_MOESM4_ESM.pdf]

Supplementary Table 3. CGI improvement among three groups.

Bumetanide groups were stratified using the median of baseline GABA/Glx ratio in the insular cortex.

CGI improvement	Control group (n,%)	Bumetanide group (n,%)	
		bumetanide. lower GABA/Glx ratios	bumetanide. higher GABA/Glx ratios
1-Very much improved	0, 0%	0, 0%	1, 4%
2-Much improved	0, 0%	10, 67%	5, 22%
3-Minimally improved	10, 71%	4, 27%	15, 65%
4-No change	4, 29%	1, 6%	2, 9%
5-Minimally worse	/	/	/

<table>
<tr><td rowspan="7">CGI improvement among three groups</td><td>degree.of.freedom</td><td colspan="2">Kruskal-Wallis.chi-squared</td><td>p-value</td></tr>
<tr><td>2</td><td colspan="2">13.64</td><td>0.0011</td></tr>
<tr><td colspan="4">post-hoc test</td></tr>
<tr><td>comparison</td><td>z</td><td>p-value</td><td>fdr-p</td></tr>
<tr><td>bumetanide. higher GABA/Glx ratios
vs. bumetanide. Lower GABA/Glx ratios</td><td>1.99</td><td>0.0468</td><td>0.0468</td></tr>
<tr><td>bumetanide. higher GABA/Glx ratios
vs. control</td><td>-2.10</td><td>0.0355</td><td>0.0532</td></tr>
<tr><td>bumetanide. lower GABA/Glx ratios
vs control</td><td>-3.69</td><td>0.0002</td><td>0.0007</td></tr>
</table>
