# Supplementary material for: Symptom improvement in children with autism spectrum disorder following bumetanide administration is associated with decreased GABA/glutamate ratios
Source: Transl Psychiatry. 2020 Jan 27;10:9. doi: 10.1038/s41398-020-0692-2 (PMC7026137; doi:10.1038/s41398-020-0692-2)
Supplement: Supplementary file 5 — Supplementary Figure 1 [file 41398_2020_692_MOESM5_ESM.docx]

**
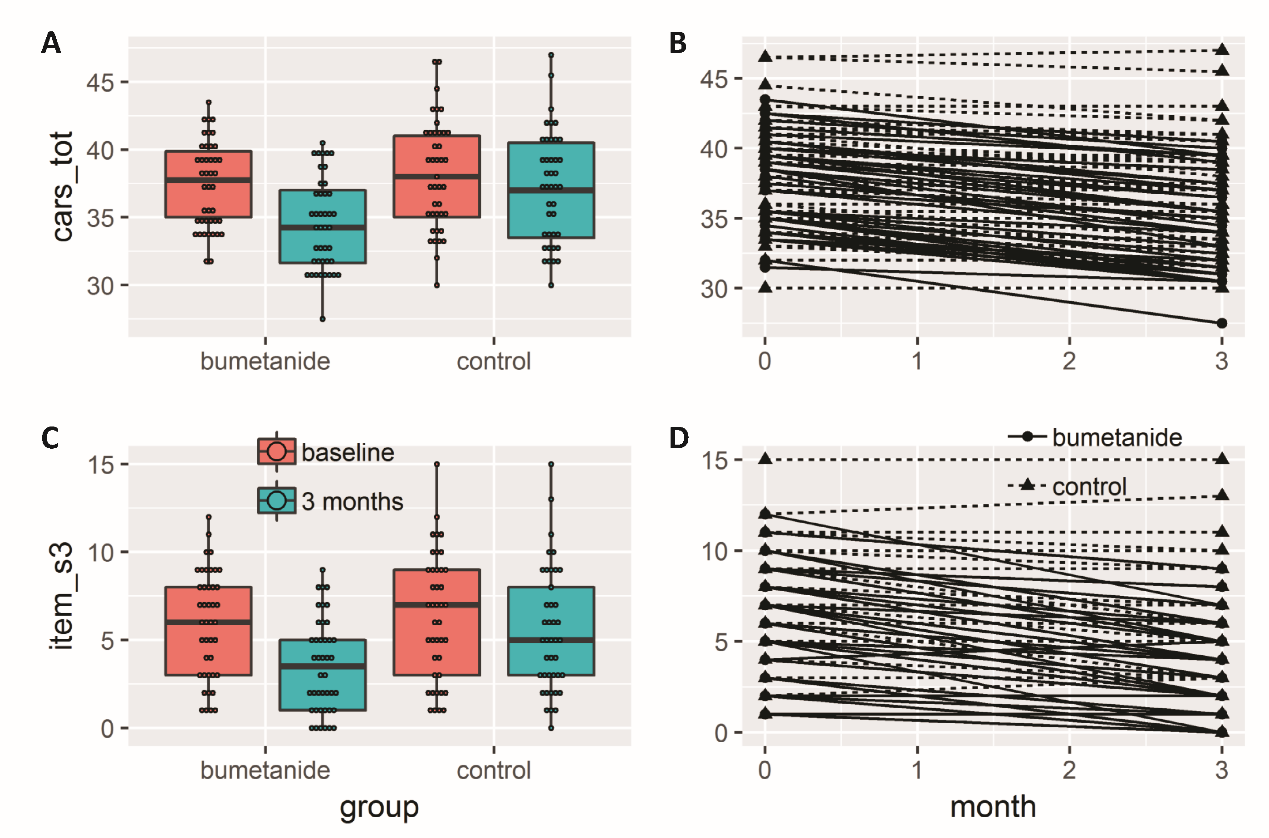
**

**Supplementary Figure 1.** **Changes in clinical symptoms after bumetanide administration.**

Group comparisons of symptom severity, indicated by both CARS total score (A) and number of items assigned a score≥3 (C), before and after treatment. Dots represent the symptom score of each subject. Trajectories of the change of symptoms severity, indicated by both CARS total score (B) and number of items assigned a score≥3 (D), before (month 0) and after (month 3) treatment. Dots represent symptom score of each subject from bumetanide group, and triangles represent the score of each subject from the control group. Abbreviations: cars_tot - total score of Childhood Autism Rating Scale, item_s3 - number of items assigned a score≥3 in CARS
